# Effective transvascular delivery of nanoparticles across the blood-brain tumor barrier into malignant glioma cells

**DOI:** 10.1186/1479-5876-6-80

**Published:** 2008-12-18

**Authors:** Hemant Sarin, Ariel S Kanevsky, Haitao Wu, Kyle R Brimacombe, Steve H Fung, Alioscka A Sousa, Sungyoung Auh, Colin M Wilson, Kamal Sharma, Maria A Aronova, Richard D Leapman, Gary L Griffiths, Matthew D Hall

**Affiliations:** 1 National Institute of Biomedical Imaging and Bioengineering, National Institutes of Health, Bethesda, Maryland 20892, USA; 2 Diagnostic Radiology Department, Clinical Center, National Institutes of Health, Bethesda, Maryland 20892, USA; 3 Imaging Probe Development Center, National Heart, Lung, and Blood Institute, National Institutes of Health, Bethesda, Maryland 20892, USA; 4 Laboratory of Cell Biology, National Cancer Institute, National Institutes of Health, Bethesda, Maryland 20892, USA; 5 Neuroradiology Department, Massachusetts General Hospital, Boston, Massachusetts 02114, USA; 6 Biostatistics, National Institute of Neurological Disorders and Stroke, National Institutes of Health, Bethesda, Maryland 20892, USA; 7 Metabolism Branch, National Cancer Institute, National Institutes of Health, Bethesda, Maryland 20892, USA; 8 Division of Biologic Drug Products, Office of Oncology Products, Center for Drug Evaluation and Research, U.S. Food & Drug Administration, Silver Spring, Maryland 20993, USA

## Abstract

**Background:**

Effective transvascular delivery of nanoparticle-based chemotherapeutics across the blood-brain tumor barrier of malignant gliomas remains a challenge. This is due to our limited understanding of nanoparticle properties in relation to the physiologic size of pores within the blood-brain tumor barrier. Polyamidoamine dendrimers are particularly small multigenerational nanoparticles with uniform sizes within each generation. Dendrimer sizes increase by only 1 to 2 nm with each successive generation. Using functionalized polyamidoamine dendrimer generations 1 through 8, we investigated how nanoparticle size influences particle accumulation within malignant glioma cells.

**Methods:**

Magnetic resonance and fluorescence imaging probes were conjugated to the dendrimer terminal amines. Functionalized dendrimers were administered intravenously to rodents with orthotopically grown malignant gliomas. Transvascular transport and accumulation of the nanoparticles in brain tumor tissue was measured *in vivo *with dynamic contrast-enhanced magnetic resonance imaging. Localization of the nanoparticles within glioma cells was confirmed *ex vivo *with fluorescence imaging.

**Results:**

We found that the intravenously administered functionalized dendrimers less than approximately 11.7 to 11.9 nm in diameter were able to traverse pores of the blood-brain tumor barrier of RG-2 malignant gliomas, while larger ones could not. Of the permeable functionalized dendrimer generations, those that possessed long blood half-lives could accumulate within glioma cells.

**Conclusion:**

The therapeutically relevant upper limit of blood-brain tumor barrier pore size is approximately 11.7 to 11.9 nm. Therefore, effective transvascular drug delivery into malignant glioma cells can be accomplished by using nanoparticles that are smaller than 11.7 to 11.9 nm in diameter and possess long blood half-lives.

## Background

Progress towards the effective clinical treatment of malignant gliomas has been hampered due to ineffective drug delivery across the blood-brain tumor barrier (BBTB), in addition to the inability to simultaneously image drug permeation through tumor tissue [1-3]. The current paradigm for treating malignant gliomas is the placement of implantable 1,3-bis (2-chloroethyl)-1-nitrosourea (BCNU, also called carmustine) wafers in the tumor resection cavity followed by administration of oral temozolomide, an alkylating agent, with concurrent radiation [4-7]. BCNU, a low molecular weight nitrosourea, is able to cross the BBTB, but is unable to accumulate within malignant glioma cells at therapeutic levels due to a short blood half-life [8]. Intra-operative placement of polymeric wafers impregnated with BCNU along the tumor resection cavity has resulted in improved patient outcomes, and significantly decreased toxicity compared to that associated with intravenous BCNU treatment [9,10]. Since this local method of BCNU delivery circumvents the BBTB and allows for sustained release of BCNU from the polymer, there are higher steady-state BCNU concentrations within the tumor resection cavity[11]. However, a major limitation of this delivery method is that the placement of the BCNU polymer wafers may only be performed at the time of initial tumor resection [12]. Temozolomide, like BCNU, has a low molecular weight and a short blood half-life which limits its ability to accumulate within malignant glioma cells [5,13].

The sizes of traditional chemotherapeutics, such as BCNU and temozolomide, are commonly reported as particle molecular weights since these particles are usually smaller than 1 nm in diameter [13]. In contrast, the sizes of nanoparticle-based therapeutics are commonly reported as particle diameters since these particles usually range between 1 and 200 nm in diameter [14,15]. Particle shapes and sizes determine how effectively particles can be filtered by the kidneys [16-18]. Spherical nanoparticles smaller than 5 to 6 nm and weighing less than 30 to 40 kD are efficiently filtered by the kidneys [17]. Spherical nanoparticles that are larger and heavier are not efficiently filtered by the kidneys; therefore, these particles possess longer blood half-lives [19]. The BBTB of malignant gliomas becomes porous due to the formation of discontinuities within and between endothelial cells lining the lumens of tumor microvessels [20]. Nanoparticles smaller than the pores within the BBTB, with long blood half-lives, could function as effective transvascular drug delivery devices for the sustained-release of chemotherapeutics into malignant glioma cells.

Even though fenestrations and gaps within the BBTB of malignant gliomas allow for unimpeded passage of low molecular weight therapeutics [21], these pores are narrow enough to prevent the effective transvascular passage of most nanoparticles [22-25]. If the upper limit of the therapeutically relevant pore size of the BBTB could be accurately determined, then intravenously administered nanoparticles, with long blood half-lives, could serve as effective drug delivery vehicles across the BBTB of malignant gliomas.

By performing intravital fluorescence microscopy of xenografted human glioma microvasculature in the mouse cranial window model, Hobbs et al. [26] observed perivascular fluorescence 24 hours following the intravenous infusion of rhodamine dye labeled liposomes of 100 nm diameters. Since then several classes of nanoparticles have been designed to be less than 100 nm in diameter for the purposes of effective transvascular drug delivery across the BBTB. These classes of nanoparticles include metal-based (i.e. iron oxide) [27], lipid-based (i.e. liposomes) [28], and biological-based (i.e. antibodies, viruses) [29,30].

Yet another class of nanoparticles are the polymer-based dendrimers [2,31]. Polyamidoamine (PAMAM) dendrimers [32] are multigenerational polymers with a branched exterior consisting of surface groups that can be functionalized with imaging [33,34], targeting [35], and therapeutic agents [35,36]. PAMAM dendrimers functionalized with low molecular weight agents remain particularly small, typically ranging between 1.5 nm (generation 1, G1) and 14 nm in diameter (generation 8, G8) [32,33]. Particle shapes are spherical and sizes are uniform within a particular generation. With each successive dendrimer generation, the number of modifiable surface groups doubles while the overall diameter increases by only 1 to 2 nm [37].

We hypothesized that the major reason for the ineffectiveness of metal-based, lipid-based and biological-based nanoparticles in traversing the BBTB of malignant gliomas is the large size of these particles relative to the physiologic pore size of the BBTB. In this work, using the RG-2 malignant glioma model [38,39], we also investigated how the transvascular transport of dendrimer nanoparticles is affected by tumor volume-related differences in the degree of BBTB breakdown.

The hyperpermeability of the BBTB of malignant gliomas results in contrast enhancement of brain tumor tissue on magnetic resonance imaging (MRI) scans following the intravenous infusion of gadolinium (Gd)-diethyltriaminepentaacetic acid (DTPA), a low molecular weight contrast agent [40,41]. To visualize the extravasation of PAMAM dendrimers across the BBTB of rodent malignant gliomas by dynamic contrast-enhanced MRI, we functionalized the exterior of PAMAM dendrimers with Gd-DTPA. Using dynamic contrast-enhanced MRI, we measured the change in contrast enhancement of malignant gliomas for up to 2 hours following the intravenous infusion of successively higher Gd-dendrimer generations up to, and including, Gd-G8 dendrimers. To verify that dendrimer size, and not dendrimer generation, is the primary determinant of particle blood half-life, we studied Gd-G4 dendrimers of two different sizes. One was a lowly conjugated Gd-G4 weighing 24.4 kD and the other was a standard Gd-G4 weighing 39.8 kD. The Gd concentration, a surrogate for the amount of Gd-dendrimer within tumor tissue, was determined by measuring the molar relaxivity of Gd-dendrimers *in vitro *in combination with the change in the blood and tissue longitudinal relaxivities (T_1_) before and after Gd-dendrimer infusion [42]. Based on comparisons of the contrast enhancement patterns of malignant gliomas for up to 2 hours, within a particular Gd-dendrimer generation as well as across Gd-dendrimer generations, we determined the physiologic upper limit of BBTB pore size.

In addition to the *in vivo *dynamic contrast-enhanced MRI experiments with Gd-dendrimers, we performed *in vitro *and *ex vivo *fluorescence microscopy experiments using rhodamine B labeled Gd- dendrimers to confirm that the impediment to the cellular uptake of functionalized dendrimers is the BBTB. The observations made in this study, using functionalized dendrimers, are to serve as a guide for designing nanoparticles that are effective at traversing the pores of the blood-brain tumor barrier and accumulating within individual glioma cells.

## Methods

### PAMAM dendrimer functionalization and characterization

Bifunctional chelating agents and gadolinium-benzyl-diethyltriaminepentaacetic acid (Gd-Bz-DTPA) functionalized PAMAM dendrimers were synthesized according to described procedures with minor modifications, as were the corresponding rhodamine-substituted conjugates [43-45]. Gd-dendrimers, with the exception of lowly conjugated Gd-G4, were prepared by using a molar reactant ratio of ≥ 2:1 bifunctional chelate to dendrimer surface amine groups. For lowly conjugated Gd-G4 a lower molar reactant ratio of 1.1:1 was used to limit conjugation. The duration of the chelation reaction for the lowly conjugated Gd-G4 was 24 hours as compared to the standard 48 hours for chelation of all other dendrimers. Rhodamine B labeled Gd-dendrimers were prepared by stirring rhodamine B isothiocyanate (RBITC) and PAMAM dendrimers at a 1:9 molar ratio of RBITC to dendrimer surface amine groups in methanol at room temperature for 12 hours. Isothiocyanate activated DTPA was then added in excess and reacted for an additional 48 hours. Gadolinium was then chelated after the removal of the *t*-butyl protective groups on DTPA. The percent by mass of Gd in each Gd-dendrimer generation was determined by elemental analysis to be: Gd-G1 (15.0%), Gd-G2 (14.8%), Gd-G3 (12.9%), lowly conjugated Gd-G4 (12.3%), standard Gd-G4 (12.0%), Gd-G5 (11.9%), Gd-G6 (11.9%), Gd-G7 (12.2%), Gd-G8 (10.2%). The Gd percent by mass for the rhodamine B Gd-dendrimers was determined to be: rhodamine B Gd-G2 (9.6%), rhodamine B Gd-G5 (9.8%), rhodamine B Gd-G8 (9.3%). Gd-G1 through Gd-G5 dendrimer molecular weights were determined by matrix assisted laser desorption/ionization time-of-flight (MALDI-TOF) mass spectroscopy (Scripps Center for Mass Spectrometry, La Jolla, CA). Gd percent by mass of the Gd-dendrimer, in its solid form, was determined with the inductively coupled plasma-atomic emission spectroscopy (ICP-AES) method (Desert Analytics, Tucson, AZ). Gd-dendrimer infusions were normalized to 100 mM with respect to Gd, while rhodamine B Gd-dendrimer infusions were normalized to 67 mM with respect to Gd, in order to guarantee proper solvation.

### *In vitro *scanning transmission electron microscopy

For *in vitro *transmission electron microscopy experiments, a 5 μl droplet of phosphate-buffer saline solution containing a sample of Gd-dendrimers from generations 5, 6, 7 or 8 was absorbed onto a 3 nm-thick carbon support film covering the copper electron microscopy grids. Lacey Formvar/carbon coated 300 meshcopper grids supporting an ultrathin 3 nm evaporated carbon film were glow-discharged an air pressure of 0.2 mbar to facilitate Gd-dendrimer adsorption. After adsorption for 2 minutes, excess Gd-dendrimer solution was blotted with filter paper. The grids were then washed 5 times with 5 μL aliquots of deionized water, and left to dry in air. Annular dark field scanning transmission electron microscope (ADF STEM) images of the Gd-dendrimers were recorded using a Tecnai TF30 electron microscope (FEI, Hillsboro, OR, USA) equipped with a Schottky field-emission gun and an in-column ADF detector (Fischione, Export, PA) [46].

### *In vitro *fluorescence experiments

For *in vitro *fluorescence experiments, RG-2 glioma cells were plated on Fisher Premium coverslips (Fisher Scientific, Pittsburgh, PA) and incubated in wells containing sterile 3 ml DME supplemented with 10% FBS (Invitrogen, Carlsbad, CA). The RG-2 glioma colonies were allowed to establish for 24 hours in an incubator set at 37°C and 5% CO_2_. Rhodamine B Gd-G2, rhodamine B Gd-G5 or rhodamine B Gd-G8 dendrimers were added to the medium by equivalent molar rhodamine B concentrations of 7.2 μM and the cells were incubated in the dark for another 4 hours. Following incubation, cells were washed 3 times with PBS, then 50 μl DAPI-Vectashield nuclear stain medium (Vector Laboratories, Burlingame, CA) was placed on the coverslips for 15 minutes. Coverslips were then inverted and mounted on Daigger Superfrost slides (Daigger, Vernon Hills, IL) and sealed into place. Confocal imaging was performed on a Zeiss 510 NLO microscope (Carl Zeiss MicroImaging, Thornwood, NY). Slides were stored in the dark while not being analyzed.

### *In vitro *magnetic resonance imaging for calculations of Gd-dendrimer molar relaxivity

Gd-dendrimer stock solution (20 μl of 100 mM) and rhodamine B Gd-dendrimer stock solution (30 μl of 67 mM) for the particular generation, used for *in vivo *imaging, was diluted using PBS into 200 μl microfuge tubes at 0.00 mM, 0.25 mM, 0.50 mM, 0.75 mM and 1.00 mM with respect to Gd. As an external control, Magnevist (Bayer, Toronto, Canada), a form of Gd-DTPA, was also diluted at the above concentrations into 200 μl microfuge tubes. The microfuge tubes were secured in level and upright positions within a plastic container filled with deionized ultra pure water. The container was placed in a 7 cm small animal solenoid radiofrequency coil (Philips Research Laboratories, Hamburg, Germany) centered within a 3.0 Tesla MRI scanner (Philips Intera; Philips Medical Systems, Andover, MA). Gd signal intensity measurements were made using a series of T_1 _weighted spin echo sequences with identical T_E _(echo time, 10 ms) but different T_R _(repetition time, 100 ms, 300 ms, 600 ms and 1200 ms). Using the measured Gd signal intensity, in addition to the known values for T_R _and T_E_, the T_1 _and equilibrium magnetization (M_0_) were calculated by non-linear regression [42]. *In vitro *and *in vivo *Gd-dendrimer molar relaxivities were assumed to be equivalent for the purposes of this work.

### Brain tumor induction and animal preparation for imaging

All animal experiments were approved by the National Institutes of Health Clinical Center Animal Care and Use Committee. Cryofrozen pathogen-free RG-2 glioma cells were obtained from the American Type Culture Collection (Rockville, MD) and cultured in sterile DME supplemented with 10% FBS and 2% penicillin-streptomycin in an incubator set at 37°C and 5% CO_2_. The anesthesia and route for all animal experiments was isoflurane by inhalation with nose cone, 5% for induction and 1 to 2% for maintenance. On experimental day 0, the head of anesthetized adult male Fischer344 rats (F344) weighing 200–250 grams (Harlan Laboratories, Indianapolis, IN) was secured in a stereotactic frame with ear bars (David Kopf Instruments, Tujunga, CA). The right anterior caudate and left posterior thalamus locations within the brain were stereotactically inoculated with RG-2 glioma cells [47]. In each location, either 20,000 or 100,000 glioma cells in 5 μl of sterile PBS were injected over 8 minutes, using a 10 μl Hamilton syringe with a 32-gauge needle. With this approach the majority of animal brains developed one large and one small glioma. On experimental days 11 to 12, brain imaging of re-anesthetized rats was performed following placement of polyethylene femoral venous and arterial cannulas (PE-50; Becton-Dickinson, Franklin Lakes, NJ), for contrast agent infusion and blood pressure monitoring, respectively. After venous cannula insertion, 50 μl of blood was withdrawn from the venous cannula for measurement of hematocrit.

### *In vivo *magnetic resonance imaging of brain tumors

All magnetic resonance imaging experiments were conducted with a 3.0 Tesla MRI scanner (Philips Intera) using a 7 cm solenoid radiofrequency coil (Philips Research Laboratories). For imaging, the animal was positioned supine, with face, head, and neck snugly inserted into a nose cone centered within the 7 cm small animal solenoid radiofrequency coil. Anchored to the exterior of the nose cone were three 200 μL microfuge tubes containing 0.00 mM, 0.25 mM and 0.50 mM solutions of Magnevist to serve as standards for measurement of MRI signal drift over time. Fast spin echo T_2 _weighted anatomical scans were performed with T_R _= 6000 ms and T_E _= 70 ms. Two different flip angle (FA) 3-D fast field echo (3D FFE) T1 weighted scans were performed with T_R _= 8.1 ms and T_E _= 2.3 ms, for quantification of Gd concentration. The first FFE scan was performed at a low FA of 3° without any contrast agent on board. The second FFE scan was performed with a high FA of 12°. For this scan, the dynamic scan, each brain volume was acquired once every 20 seconds, for 1 to 2 hours. During the beginning of the dynamic scan, three to five baseline brain volumes were acquired prior to Gd-dendrimer infusion. Gd-dendrimers were infused at doses of 0.03, 0.06 or 0.09 mmol Gd/kg bw depending on the experiment. Gd-dendrimer was infused as a bolus over 1 minute in order to accurately measure the contrast agent dynamics in blood during the bolus. Following completion of the 1 or 2 hour dynamic contrast-enhanced MRI scan, another 15 minute dynamic contrast-enhanced MRI scan was performed during which Magnevist was infused at a dose of 0.30 mmol Gd/kg bw over 1 minute. Tumor regions of interest were drawn based on the Magnevist dynamic scan data.

### Dynamic contrast-enhanced MRI data analyses and pharmacokinetic modeling

Imaging data was analyzed using the Analysis of Functional NeuroImaging (AFNI; ) software suite and its native file format [48]. Motion correction was performed by registering each volume of the dynamic high FA scan to its respective low FA scan. Alignments were performed using Fourier interpolation. A baseline T_1 _without contrast (T_10_) map was generated by solving equation 1 (the steady-state for incoherent signal after neglecting T_2_* effects) voxel-by-voxel for T_1_, at both low and high FA's, before contrast was infused [42].

(1) $$S=\frac{M_{0}(1-E_{1})\sin\theta }{1-E_{1}\cos\theta }$$

where

(2) $$E_{1}=\exp\left(-\frac{T_{R}}{T_{1}}\right)$$

After determining the T_10 _value at each voxel, T_1 _map was calculated using equations 1 and 2 for each voxel of each dynamic image during the high FA scan after contrast infusion [42]. Datasets were converted to Gd concentration space [42]. Whole tumor regions of interest were drawn on the basis of the dynamic contrast enhancement pattern of tumor tissue observed following the infusion of Magnevist. These data were important for the drawing of accurate whole tumor regions of interest for minimally enhancing gliomas, especially for all malignant gliomas within the 0.03 mmol Gd/kg bw Gd-dendrimer dose category and those in the 0.09 mmol Gd/kg bw Gd-G8 dendrimer dose sub-category. Normal brain regions of interest were spherical 9 mm^3 ^volumes in the left anterior caudate.

The pharmacokinetic properties of Gd-G1 through lowly conjugated Gd-G4 dendrimers were modeled using the dynamic contrast-enhanced MRI data from the groups of animals receiving 0.09 mmol Gd/kg bw Gd-dendrimer infusions. The change in blood Gd-dendrimer concentration over time was obtained by selecting 2 to 3 voxels within the superior sagittal sinus, a large caliber vein that is minimally where influenced by in-flow and partial volume averaging effects. Since the transit time of blood movement between an artery and a vein within the brain is approximately 4 seconds, while the image acquisition rate was once every 20 seconds, the superior sagittal sinus was used for generation of the vascular input function for pharmacokinetic modeling [41]. Animal brains from which an optimal vascular input function could not be obtained were excluded from being analyzed by pharmacokinetic modeling. The voxels chosen had peak blood Gd concentrations closest to the calculated initial Gd-dendrimer volume of distribution, based on the blood volume of a 250 gram rat being 14 ml [49]. Blood concentration was converted to plasma concentration by correcting for the hematocrit (Hct) as shown in equation 3 [40].

(3) $$C_{\text{p}}=\frac{C_{\text{b}}}{1-\text{Hct}}$$

The 2-compartment 3-parameter generalized kinetic model (equation 4) [40,50] was employed for pharmacokinetic modeling by performing voxel-by-voxel nonlinear regression over all time points.

(4) $$C_{\text{t}}(t)=v_{\text{p}}C_{\text{p}}(t)+K^{\text{trans}}\int_{0}^{t}C_{\text{p}}(\tau )\exp\left(\frac{-K^{\text{trans}}(t-\tau )}{v_{\text{e}}}\right)d\tau$$

Constraints on the parameters were set between 0 and 1 calling on 10,000 iterations. Least squares minimizations were performed by implementing the Nelder-Mead simplex algorithm. Prior to statistical analysis, voxels with poor fits or non-physiologic parameters were censored.

### *Ex vivo *fluorescence microscopy and histological staining of brain tumor sections

Six additional rats received 0.06 mmol Gd/kg bw of rhodamine B Gd-G5 and two additional rats received 0.06 mmol Gd/kg bw of rhodamine B Gd-G8. Subsequent to the standard 2 hour dynamic contrast-enhanced MRI study, the brains of these animals were harvested and snap-frozen. On the day of cryosectioning, two 10 μm sections of tumor bearing brain were cut onto each Daigger Superfrost slide with a Leica Cryotome (Leica, Bensheim, Germany). The first of two slides was prepared for fluorescence microscopy by application of DAPI-Vectashield nuclear stain medium and coversliping. Confocal imaging was performed on a Zeiss 510 NLO microscope. The second slide was stained with Hematoxylin and Eosin for visualization of tumor histology.

### Statistical analysis for pharmacokinetic modeling

Vascular parameter pharmacokinetic values for individual tumor voxels were averaged in order to yield one value per parameter per tumor per rat, with tumors within a rat being treated as correlated. On the basis of the range of individual tumor volumes within Gd-G1, Gd-G2, Gd-G3 and lowly conjugated Gd-G4 dendrimer study groups, a dichotomous variable for tumor size was generated by using 50 mm^3 ^as the cut-off between large and small tumors. Multivariate analysis of variance (MANOVA) models were used to examine the effect of dendrimer generation and tumor size. Prior to the MANOVA, it determined that there was no interaction between dendrimer generation and tumor size on any of the three parameters. The covariance structure was considered to be compound symmetric and the Kenward-Roger degrees of freedom method was used. Post-hoc comparisons between lowly conjugated Gd-G4 and each of the other generations were conducted. The significant *P*-values we report are following Bonferroni correction for multiple comparisons. Analyses were implemented in SAS PROC Mixed (SAS Institute Inc., Cary, North Carolina) with α = 0.05.

## Results

### Physical properties of naked PAMAM and Gd-PAMAM dendrimer generations

**Figure 1:**
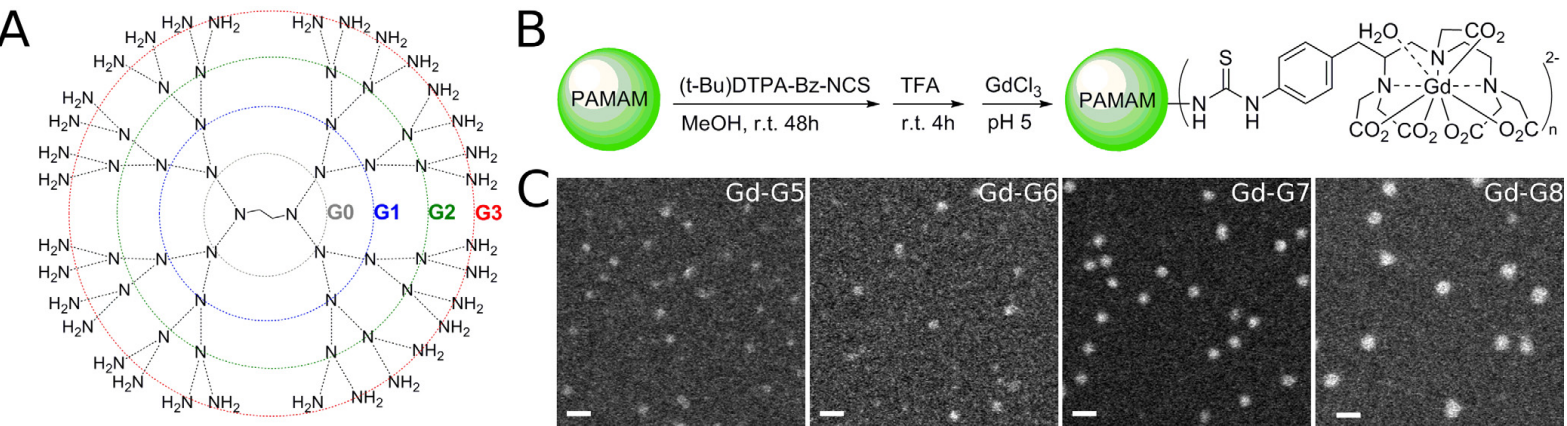
**Synthesis of Gd-dendrimers and transmission electron microscopy of higher generation Gd-dendrimers**. A) A two-dimensional representation of naked polyamidoamine dendrimers up until generation 3 showing ethylenediamine core. B) The naked dendrimer has a cationic exterior. Functionalizing the terminal amine groups with Gd-diethyltriaminepentaacetic acid (charge -2) neutralizes the positive charge on the dendrimer exterior. C) Annular dark-field scanning transmission electron microscopy images of Gd-G5, Gd-G6, Gd-G7, and Gd-G8 dendrimers adsorbed onto an ultrathin carbon support film. Scale bar = 20 nm.

The physical properties of naked PAMAM dendrimers (Starburst G1–G8, ethylenediamine core; Sigma-Aldrich, St. Louis, MO) and Gd-PAMAM dendrimers are detailed in  table 1. Naked full generation PAMAM dendrimers are cationic due to the presence of amine groups on the dendrimer exterior for conjugation (Figure 1A). With each successive dendrimer generation both the molecular weight and number of terminal amines doubles. Conjugation of Gd-DTPA (charge -2, molecular weight ~0.7 kD) to the surface amine groups of naked PAMAM dendrimers neutralizes the positive charge on dendrimer exterior (Figure 1B). The molecular weight increase of the naked dendrimer to that of the Gd-DTPA conjugated dendrimer is proportional to the percent conjugation of Gd-DTPA (Table 1). The percent conjugation of lowly conjugated Gd-G4 dendrimers was 29.8% whereas that of standard Gd-G4 dendrimers was 47.5% (Table 1). The constants of proportionality required for calculation of Gd concentration, also known as Gd-dendrimer molar relaxivities, ranged between 7.8 and 12.2 s/mM (Table 1).

**Table 1:** Table 1 - Physical properties of PAMAM and Gd-PAMAM dendrimer generations

Dendrimer generation (G)	No. terminal amines	Naked PAMAM molecular weight^# ^(kD)	Gd-PAMAM molecular weight^† ^(kD)	Gd-DTPA conjugation (%)	Molar relaxivity^&^(s/mM)
G1	8	1.43	5.63	67.1	9.8
G2	16	3.26	11.2	65.9	10.1
G3	32	6.91	18.6	47.7	10.4
Lowly conjugated G4	64	14.2	24.4	29.8	7.8
Standard G4	64	14.2	39.8	47.5	12.2
G5	128	28.8	79.8	47.2	10.9
G6	256	58.0	133	39.9	10.6
G7	512	116	330^‡^	50.0	10.3
G8	1024	233	597^‡^	37.8	9.4

^#^obtained from Dendritech, Inc.

^†^measured by MALDI-TOF MS unless noted otherwise

^‡^measured by ADF STEM

^&^molar relaxivity of Gd-DTPA measured to be 4.1

Since the sizes of hydrated dendrimer generations, measured by small-angle X-ray scattering (SAXS) [51] and small-angle neutron scattering (SANS) [52], are similar to the sizes of respective dehydrated dendrimer generations measured by TEM [37], we were able to use ADF STEM to image Gd-G5 and higher generation Gd-dendrimers: these Gd-dendrimer generations possessed masses heavy enough to be visualized by ADF STEM [46,53]. ADF STEM images of Gd-G5 through Gd-G8 dendrimers demonstrated uniformity in particle size, shape and density within any particular dendrimer generation (Figure 1C). These images also confirmed a small increase of approximately 2 nm in particle diameter between successive generations. The diameters of sixty Gd-G7 and Gd-G8 dendrimers were measured. The average diameter of our Gd-G7 dendrimers was 11.0 ± 0.7 nm and that of Gd-G8 dendrimers was 13.3 ± 1.4 nm (mean ± standard deviation).

### Effect of Gd-dendrimer dose on particle extravasation across the blood-brain tumor barrier

The transvascular transport of Gd-G1 through Gd-G8 dendrimers across pores of the BBTB and accumulation within brain tumor tissue were studied at Gd-dendrimer doses of 0.03 mmol Gd/kg bw and 0.09 mmol Gd/kg bw. The 0.03 mmol Gd/kg bw dose is the standard intravenous Gd-dendrimer dose for pre-clinical imaging with Gd-dendrimers [33]. For each Gd-dendrimer generation, the amount of Gd-dendrimer infused at the 0.03 mmol Gd/kg bw and 0.09 mmol Gd/kg bw doses is shown in the supplementary table (Additional file 1).

**Figure 2:**
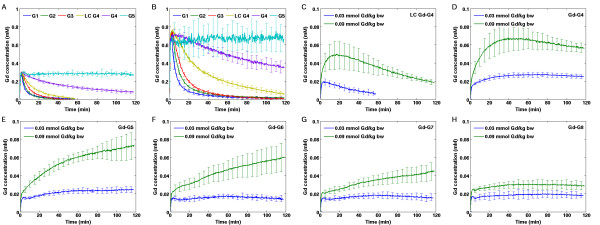
**Gd concentration within blood and glioma tissue over time following intravenous Gd-dendrimer infusions at doses of 0.03 mmol Gd/kg bw and 0.09 mmol Gd/kg bw**. A) Blood concentrations of Gd-dendrimers measured in the superior sagittal sinus following 0.03 mmol Gd/kg bw infusion. Gd-G1 (n=6), Gd-G2 (n=5), Gd-G3 (n=5), and lowly conjugated Gd-G4 (n=5) dendirmers imaged for 1 hour. Standard Gd-G4 (n=6), Gd-G5 (n=6), Gd-G6 (n=5), Gd-G7 (n=6), and Gd-G8 (n=5) dendrimers imaged for 2 hours. Error bars represent standard deviations. B) Blood concentrations of Gd-dendrimers measured in the superior sagittal sinus following 0.09 mmol Gd/kg bw infusion.  Gd-G1 (n=4), Gd-G2 (n=6), Gd-G3 (n=6), lowly conjugated Gd-G4 (n=4), standard Gd-G4 (n=6), Gd-G5 (n=6), Gd-G6 (n=5), Gd-G7 (n=5), and Gd-G8 (n=6). Blood concentrations of Gd-G6, Gd-G7, and Gd-G8 dendrimers not shown for clarity. C) At both doses, lowly conjugated Gd-G4 dendrimers (molecular weight 24.4 kD) remain for a short period of time within the extravascular tumor space. 0.03 mmol Gd/kg bw dose n=5, 0.09 mmol Gd/kg bw dose n=4. D) At both doses, standard Gd-G4 dendrimers (molecular weight 39.8 kD) remain for longer within the extravascular tumor space. 0.03 mmol Gd/kg bw dose n=6, 0.09 mmol Gd/kg bw dose n=6. E) At both doses, Gd-G5 dendrimers accumulate within the extravascular tumor space. 0.03 mmol Gd/kg bw dose n=6, 0.09 mmol Gd/kg bw dose n=6. F) At the 0.03 mmol Gd/kg bw dose (n=5), Gd-G6 dendrimers do not extravasate out of tumor microvasculature. At the 0.09 mmol Gd/kg bw dose (n=5), Gd-G6 dendrimers extravasate. G) At the 0.03 mmol Gd/kg bw dose (n=6), Gd-G7 dendrimers do not extravasate. At the 0.09 mmol Gd/kg bw dose (n=5), Gd-G7 dendrimers extravasate. H) Irrespective of dose, Gd-G8 dendrimers do not extravasate out of brain tumor microvasculature. 0.03 mmol Gd/kg bw dose n=5, 0.09 mmol Gd/kg bw dose n=6. In panels C through H, Gd tumor concentrations and standard deviations shown are weighted for total tumor volume.

**Figure 3:**
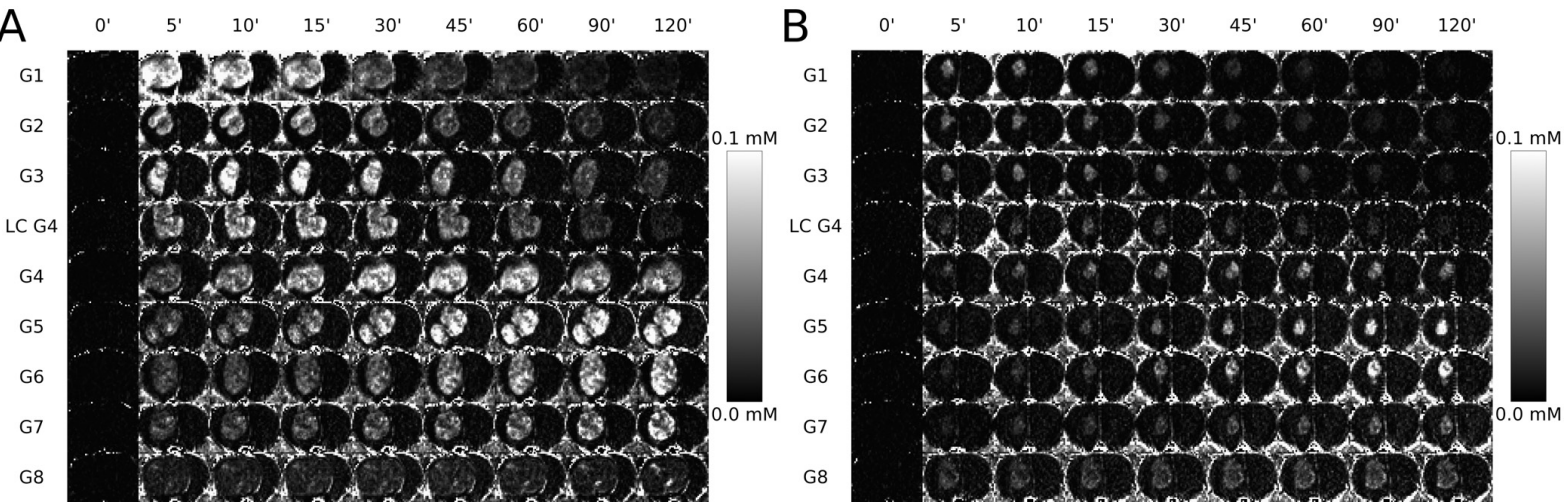
**Gd concentration maps showing Gd-dendrimer distribution within the largest and smallest gliomas of each generation over time**. A) Gd-G5, Gd-G6, and Gd-G7 dendrimers slowly accumulate within the extravascular tumor space of the largest RG-2 gliomas within the size range of tumors in the study. Gd-G8 dendrimers remain intravascular. The volume, in mm^3^, for each tumor shown is 104 (Gd-G1), 94 (Gd-G2), 94 (Gd-G3), 162 (lowly conjugated Gd-G4), 200 (standard Gd-G4), 230 (Gd-G5), 201 (Gd-G6), 170 (Gd-G7), and 289 (Gd-G8). B) Gd-G5 and G6 dendrimers still slowly accumulate within tumor tissue of the smallest RG-2 gliomas, which have a minimally compromised blood-brain tumor barrier. Gd-G7 dendrimers are impermeable to the BBTB of the smallest RG-2 gliomas and remain intravascular. Gd-G8 dendrimers continue to be impermeable to the blood-brain tumor barrier of the smallest RG-2 gliomas. The volume, in mm^3^, for each tumor shown is 27 (Gd-G1), 28 (Gd-G2), 19 (Gd-G3), 24 (lowly conjugated Gd-G4), 17 (standard Gd-G4), 18 (Gd-G5), 22 (Gd-G6), 24 (Gd-G6), and 107 (Gd-G8). Each animal received an intravenous 0.09 mmol Gd/kg bw.

At the 0.03 mmol Gd/kg bw dose, Gd-G1 through Gd-G5 dendrimers extravasated across the BBTB into the extravascular tumor space (Additional file 2; Figure 2C, 2D, and 2E). At the 0.03 mmol Gd/kg bw dose, Gd-G6, Gd-G7 and Gd-G8 dendrimers did not extravasate across the BBTB (Figure 2F, 2G, and 2H). At the 0.09 mmol Gd/kg bw dose, Gd-G1 through Gd-G6 dendrimers extravasated across the BBTB into the extravascular tumor space (Additional file 2; Figure 2C through 2F). At the 0.09 mmol Gd/kg bw dose, we found that Gd-G7 dendrimers did not extravasate across the less defective BBTB of the smallest gliomas within the size range of brain tumors in our study (Figure 3B). In the case of the largest RG-2 gliomas within the size range of brain tumors in our study, Gd-G7 dendrimers extravasated across the more defective BBTB as shown in Figure 3A. At both doses, irrespective of the degree of BBTB defectiveness related to tumor size, we found that Gd-G8 dendrimers are impermeable to the BBTB and remain within brain tumor microvasculature (Figure 2H and Figure 3).

### Effect of Gd-dendrimer dose and blood half-life on particle accumulation within brain tumor tissue

At both doses, we found that Gd-G1 through lowly conjugated Gd-G4 dendrimers possess short blood half-lives compared to Gd-dendrimers of higher generations. The blood concentration profile of lowly conjugated Gd-G4 dendrimers was similar to the profiles of Gd-G1, Gd-G2 and Gd-G3 dendrimers suggesting rapid clearance from blood circulation. Standard Gd-G4 dendrimers had a longer blood half-life than lowly conjugated Gd-G4 dendrimers due to the increase in size associated with an approximately 15 kD increase in molecular weight (Figure 2A and 2B, Table 1). At both doses, Gd-G5 through Gd-G8 dendrimers rapidly attained peak blood concentrations and then maintained steady state levels for at least 2 hours following the infusion (Figure 2A and 2B).

At both doses, Gd-G1 through lowly conjugated Gd-G4 dendrimers temporarily accumulated within the extravascular tumor space before wash-out due to short blood half-lives (Additional file 2 and Figure 2C). At both doses, standard Gd-G4 dendrimers remained within the tumor extravascular space longer than the lowly conjugated Gd-G4 dendrimers (Figure 2D). At both doses, Gd-G5 dendrimers demonstrated a steady rate of accumulation over two hours, although, at the 0.09 mmol Gd/kg bw dose the accumulation was faster over the first hour (Figure 2E). At the 0.03 mmol Gd/kg bw dose Gd-G6 dendrimers did not accumulate. At the 0.09 mmol Gd/kg bw dose, irrespective of tumor size, Gd-G5 and Gd-G6 dendrimers continued to accumulate slowly over 2 hours in all RG-2 gliomas (Figure 2 and Figure 3). Gd-G1 through Gd-G8 dendrimers remained within the microvasculature of normal brain tissue and, as a result, normal brain tissue Gd concentration curves mirrored Gd concentration curves of the superior sagittal sinus (Additional file 3).

### Effect of Gd-dendrimer size on transvascular flow rate and particle distribution within brain tumor tissue

We investigated the relationship between lower Gd-dendrimer generations and tumor volume to the particle transvascular flow rate (permeability, *K*^trans^) and distribution in the extravascular extracellular tumor volume (fractional extravascular extracellular volume, *v*_e_) using the 2-compartment 3-parameter generalized kinetic model. The third calculated vascular parameter was the tumor fractional plasma volume (*v*_p_) [40,50]. We were able to successfully model the blood and tissue pharmacokinetic behavior of only Gd-G1 through lowly conjugated Gd-G4 dendrimers since these lower Gd-dendrimer generations possess short blood half-lives and, therefore, remain predominantly within the extracellular tumor space. Higher Gd-dendrimer generations do not remain in the extracellular tumor space, but instead accumulate within glioma cells, defying the fundamental assumption of dynamic contrast-enhanced MRI-based modeling that an agent remain extracellular [40].

**Figure 4:**
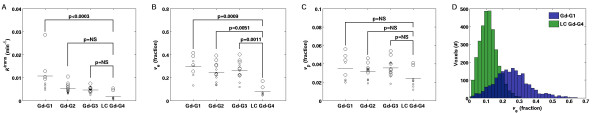
**Modeled pharmacokinetic parameters of lower generation Gd-dendrimers**. A) The increase in Gd-dendrimer generation and size from that of Gd-G1 to that of lowly conjugated Gd-G4 results in a decrease in particle transvascular flow rate (K^trans^). Large tumors have higher K^trans^ values. B) Lowly conjugated Gd-G4 dendrimer distribution within the glioma extravascular extracellular space (v_e_) is influenced to the greatest extent by the decrease in K^trans^. Large tumors have higher v_e_ values. C) Fractional plasma volume (v_p_) within glioma vasculature is maintained across dendrimer generations. Large tumors have higher v_p_ values. Large circles (Gd-G1 n= 4, Gd-G2 n=6, Gd-G3 n=7, and Gd-G4 n=2) represent large tumors (> 50 mm^3^), small circles (Gd-G1 n=4, Gd-G2 n=6, Gd-G3 n=5, and Gd-G4 n=6) represent small tumors (< 50 mm^3^), horizontal bars represent mean of observations weighted with respect to individual tumor volumes. Shown are Bonferroni corrected p-values from the nine post hoc comparisons for the three parameters, NS = not significant. D) There a more widespread distribution of Gd-G1 particles within the extravascular extracellular tumor space as shown by the greater range of v_e_ values; whereas, there is a more focal distribution of lowly conjugated Gd-G4 dendrimers as shown by the lower range of v_e_ values. Shown are voxels surviving censorship. Tumor volumes, in mm3, for tumors shown are 104 (Gd-G1) and 162 (lowly conjugated Gd-G4).

Based on the range of tumor sizes within the Gd-G1 through lowly conjugated Gd-G4 dendrimer groups, RG-2 gliomas were classified as large (> 50 mm^3^) and small (< 50 mm^3^). Irrespective of tumor size, we found significant differences between the four dendrimer generations with respect to particle transvascular flow rates (F_3,15.7 _= 11.61; Bonferroni corrected p = 0.0009, MANOVA) and distribution within the extravascular extracellular tumor volume (F_3,16.1 _= 8.26; Bonferroni corrected p = 0.0045, MANOVA), but not the tumor fractional plasma volume (F_3,16.3 _= 1.24; *P *= NS, MANOVA) (Figure 4A, 4B, and 4C). The transvascular flow rate of lowly conjugated Gd-G4 dendrimers was significantly lower compared to that of Gd-G1 dendrimers. As a consequence, lowly conjugated Gd-G4 dendrimers were focally distributed within the extravascular extracellular tumor volume (Figure 4A, 4B, and 4D). The vascular plasma volume was not significantly different between tumor populations within the four different dendrimer generations (Figure 4C). Irrespective of dendrimer generation, we found that large tumors had higher values of transvascular flow rates (F_1,34.6 _= 10.83; Bonferroni corrected p = 0.0069, MANOVA), fractional extravascular extracellular volume (F_1,22.5 _= 50.76; Bonferroni corrected p < 0.0003, MANOVA) and fractional plasma volume (F_1,27.9 _= 20.49; Bonferroni corrected p = 0.0003, MANOVA) than small tumors.

### Glioma cell uptake of fluorescent Gd-dendrimer generations *in vivo *versus *ex vivo*

**Figure 5:**
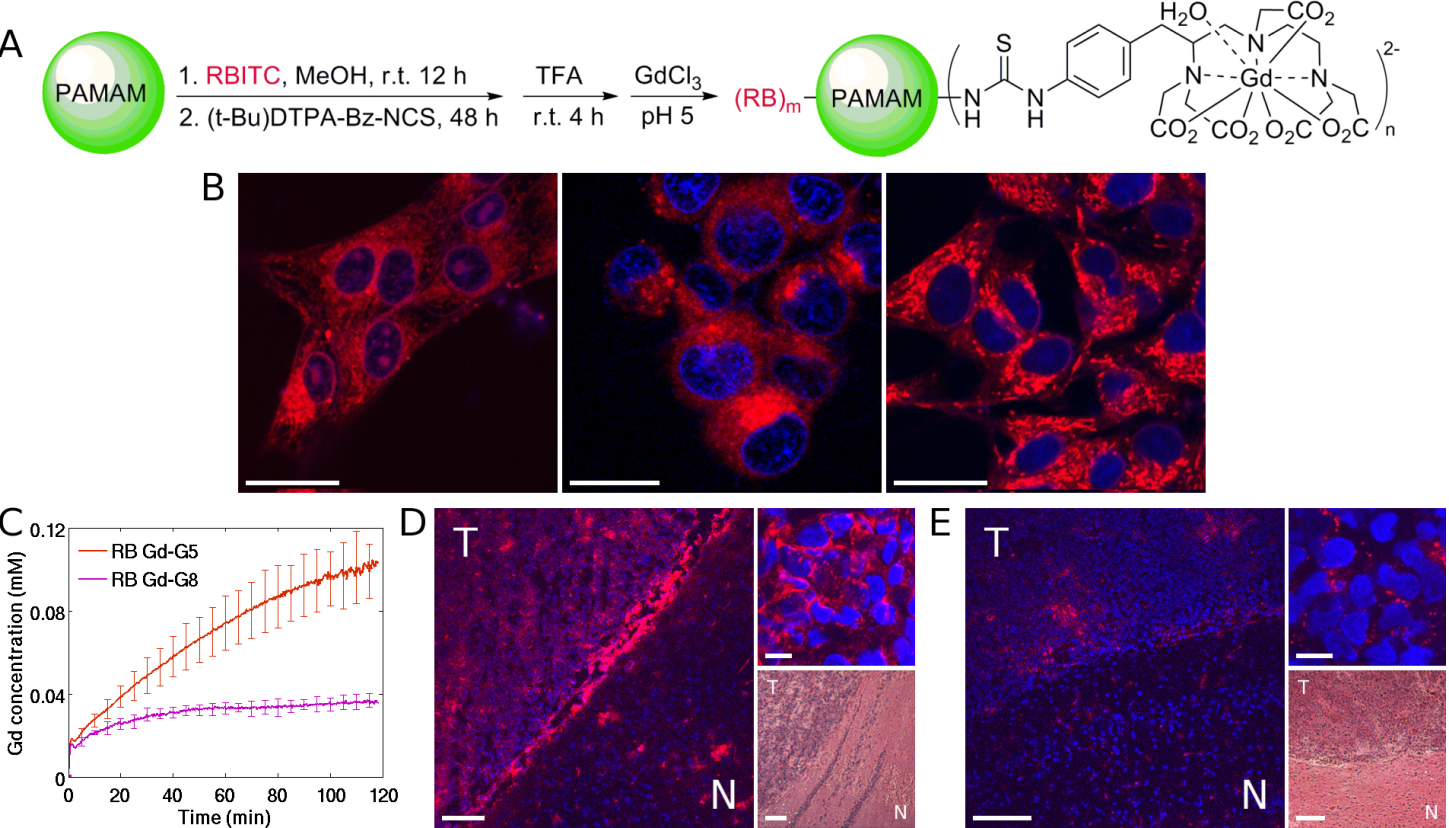
**Fluorescence microscopy of glioma cell uptake of rhodamine B labeled Gd-dendrimer generations *in vivo *versus *ex vivo***. A) Synthetic scheme for production of rhodamine B (RB) labeled Gd-polyamidoamine dendrimers. The naked polyamidoamine dendrimer is first reacted with rhodamine B and then with Gd-DTPA. B) As shown by fluorescence microscopy *in vitro*, rhodamine B Gd-G2, rhodamine B Gd-G5, and rhodamine B Gd-G8 accumulate in glioma cells. Rhodamine B Gd-G2 dendrimers enter RG-2 glioma cells, and in some cases, the nucleus (left). Rhodamine B Gd-G5 dendrimers enter the cytoplasm of RG-2 glioma cells, but do not localize within the nucleus (middle). Rhodamine B Gd-G8 dendrimers enter RG-2 glioma cells *in vitro* (right). Shown are merged confocal images of blue fluorescence from DAPI-Vectashield nuclear (DNA) stain and red fluorescence from rhodamine B labeled Gd-dendrimers. Scale bars = 20 µm. C) At 2 hours dynamic contrast-enhanced MRI shows substantial extravasation of rhodamine B Gd-G5 dendrimers and some extravasation of rhodamine B Gd-G8 dendrimers. Rhodamine B Gd-G5 n=6, rhodamine B Gd-G8 n=2. D) Low power fluorescence microscopy *ex vivo* of brain tumor and normal brain surrounding tumor shows that there is substantial accumulation of rhodamine B Gd-G5 dendrimers within tumor tissue (left, T = tumor, N = normal, scale bar = 100 µm). High power shows subcellular localization within malignant glioma cells (upper right, scale bar = 20 µm). Hemotoxylin and Eosin stain of tumor and surrounding brain (lower right, scale bar = 100 µm). Tumor volume is 31 mm^3^. E) Also shown by low power fluorescence microscopy *ex vivo* is some accumulation of rhodamine B Gd-G8 dendrimers within brain tumor tissue (left, T = tumor, N = normal, scale bar = 100 µm). High power confirms minimal subcellular localization within glioma cells (upper right, scale bar = 20 µm). Hematoxylin and Eosin stain of tumor and surrounding brain (lower right, scale bar = 100 µm). Tumor volume is 30 mm^3^.

We performed fluorescence microscopy experiments *in vitro *to confirm that the limitation to particle entry into glioma cells is not at the cellular level. Rhodamine B labeled Gd-G2, rhodamine B labeled Gd-G5, and rhodamine B labeled Gd-G8 dendrimers were synthesized as representative examples of the Gd-G1 through Gd-G8 dendrimer series. The synthetic scheme of rhodamine B Gd-dendrimers is shown in Figure 5A. The physical properties of rhodamine B Gd-G2, rhodamine B Gd-G5 and rhodamine B Gd-G8 dendrimers are displayed in Additional file 4. The physical properties of the rhodamine B dendrimers were similar to those of the Gd-G2, Gd-G5, and Gd-G8 dendrimers. RG-2 glioma cells were imaged 4 hours after addition of rhodamine B Gd-G2, rhodamine B Gd-G5 or rhodamine B Gd-G8 dendrimers into the culture media at equimolar concentrations with respect to rhodamine B. All three Gd-dendrimer generations accumulated within RG-2 glioma cells (Figure 5B). In addition, rhodamine B Gd-G2 dendrimers in some cases were observed to localize within cell nuclei (Figure 5B, left). Rhodamine B Gd-G8 dendrimers localize within glioma cells as readily as rhodamine B Gd-G5 dendrimers indicating that cellular uptake was not the barrier to the accumulation of higher generation Gd-dendrimers within glioma cells.

We conducted additional dynamic contrast-enhanced MRI experiments with correlative fluorescence microscopy of glioma specimens *ex vivo *to confirm that permeable functionalized dendrimers with long blood half-lives accumulate in glioma cells. The infusion dose for rhodamine B Gd-G5 and rhodamine B Gd-G8 dendrimers was 0.06 mmol Gd/kg bw. Rhodamine B labeling of Gd-G5 dendrimers resulted in the enhanced extravasation of rhodamine B Gd-G5 dendrimers across the BBTB and rhodamine B labeling of Gd-G8 dendrimers resulted in some extravasation of rhodamine B Gd-G8 dendrimers across the BBTB, as shown by the dynamic contrast-enhanced MRI concentration curves in Figure 5C. There was substantial accumulation of rhodamine B Gd-G5 dendrimers within tumor tissue cells as shown by fluorescence microscopy *ex vivo *(low power, Figure 5D, left). The subcellular localization of rhodamine B Gd-G5 dendrimers in tumor tissue was similar to what was observed in cultured RG-2 glioma cells (high power, Figure 5D, top right). There was some accumulation of rhodamine B Gd-G8 dendrimers within tumor tissue (Figure 5E, left). The subcellular localization of rhodamine Gd-G5 dendrimers in tumor tissue was minimal to what was observed in cultured glioma cells (Figure 5E, top right). There was a small amount of extravasation of rhodamine B Gd-G5 and rhodamine B Gd-G8 dendrimer across the normal blood-brain barrier beginning approximately 1 hour following intravenous infusion, as shown by dynamic contrast-enhanced MRI in Additional file 5.

## Discussion

Effective transvascular delivery of therapeutics into malignant glioma cells remains challenging. Although conventional low-molecular weight chemotherapeutics can easily cross the pores within the BBTB of malignant gliomas [21,54], these drugs do not achieve and maintain effective steady state concentrations within malignant glioma cells because of short blood half-lives.

Ultrastructural studies of brain tumor microvasculature have shown that fenestrations and gaps exist within the BBTB ranging from 40 to 90 nm and 100 to 250 nm, respectively [20,55]. Using intravital microscopy, Hobbs et al. [26] have reported that there is primarily perivascular fluorescence in xenografted human malignant gliomas 24 hours after the intravenous infusion of long-circulating rhodamine labeled liposomes 100 nm in diameter. Using MRI, Moore et al. [25] and Muldoon et al. [56] have reported that there is minimal contrast enhancement of rodent gliomas 24 hrs after the intravenous infusion of various long-circulating dextran coated iron oxide (also known as LCDIO) nanoparticles with a mean diameter of 20 nm [57,58]. These findings indicate that the therapeutically relevant upper limit of the BBTB pore size should range between 20 nm and 100 nm. However, the effective transvascular delivery of nanoparticle-based drug carriers across the BBTB into malignant glioma cells has remained elusive, to date. We reasoned that the physiologic upper limit of BBTB pores size would be less than 20 nm in diameter. We were aware that PAMAM dendrimers are particularly small multigenerational nanoparticles of uniform sizes within a generation [31,37]. Functionalized PAMAM dendrimer particle sizes typically range between 1.5 nm (G1) and 14 nm (G8) in diameter following the conjugation of low molecular weight imaging compounds to the dendrimer exterior [33]. In order to probe the physiologic upper limit of BBTB pore size in RG-2 malignant glioma microvasculature with dynamic contrast-enhanced MRI, we functionalized PAMAM dendrimers G1 through G8 with Gd-DTPA (charge -2) [33,34,45]. As a result of the conjugation of Gd-DTPA to approximately half of the surface amine groups, the positive surface charge on the PAMAM dendrimer exterior was neutralized. In order to confirm that the barrier to cellular entry of Gd-dendrimers is at the level of the BBTB, and that permeable functionalized dendrimers with long blood half-lives can accumulate in malignant glioma cells, we used rhodamine B labeled Gd-dendrimers for fluorescence imaging *in vitro *and *ex vivo*. Based on these studies, we report here that the physiologic upper limit of BBTB pore size ranges between approximately 11.7 and 11.9 nm. We also report that permeable functionalized dendrimers with long blood half-lives can accumulate within glioma cells.

We observed that there was virtually no contrast enhancement of malignant glioma tissue over 2 hours on dynamic-contrast enhanced MRI following the intravenous infusion of Gd-G8 dendrimers. We found this to be the case at both Gd-dendrimer doses investigated, one being the standard 0.03 mmol Gd/kg bw dose for pre-clinical dynamic contrast-enhanced MRI and the other being 0.09 mmol Gd/kg bw [33]. These dynamic contrast-enhanced MRI findings demonstrate that Gd-G8 dendrimers are larger than the upper limit of the physiologic pore size of the BBTB of RG-2 gliomas. Using ADF STEM, we measured the diameters of a population of our Gd-G8 dendrimers to be 13.3 ± 1.4 nm (mean ± standard deviation) and that of Gd-G7 dendrimers to be 11.0 ± 0.7 nm. Based on these ADF STEM data, the range of the physiologic upper limit of BBTB pore size in RG-2 malignant gliomas is between 11.7 and 11.9 nm.

To confirm that the limitation to functionalized G8 dendrimer entry is not at the cellular level, we performed fluorescence microscopy of cultured RG-2 glioma cells following the application of rhodamine B labeled Gd-dendrimers to the media. We found that rhodamine B labeled Gd-G2, -G5 and -G8 dendrimers accumulated in the cytoplasm of all RG-2 glioma cells; however, we found it particularly interesting that, in some cases, rhodamine B labeled Gd-G2 dendrimers also accumulated in the RG-2 glioma cell nuclei. This finding suggests that it may also be possible for other smaller nanoparticles (i.e. molecular weight ≤ 11.2 kD) to cross nuclear pores.

Irrespective of dose, we found that Gd-G1, Gd-G2, Gd-G3 and lowly conjugated Gd-G4 (molecular weight 24.4 kD) dendrimers had short blood half-lives because particle sizes of these lower generation Gd-dendrimers are small enough that particles can be efficiently filtered by the kidneys [17]. Therefore, Gd-G1 through lowly conjugated Gd-G4 dendrimers only remain temporarily within the tumor extravascular extracellular space. We also found that as the Gd-dendrimer generation and particle size increased, the transvascular flow (*K*^*trans*^) rate decreased; and that the lower transvascular flow rate of lowly conjugated Gd-G4 dendrimers resulted in the more focal distribution of particles within brain tumor tissue. Therefore, since lower generation dendrimers have short blood half-lives, the transvascular flow rate across the BBTB is the primary determinant of how widespread particle distribution was within the extravascular extracellular tumor space. These findings suggest that nanoparticles with higher molecular weights, *yet *particle sizes small enough to *still *be effectively filtered by the kidneys, do not remain within the extravascular tumor space sufficiently long to effectively permeate through tumor tissue. Therefore, such nanoparticles would remain within close proximity of tumor microvessels, and would not reach malignant glioma cells located within tumor regions that are poorly vascularized.

We found that standard Gd-G4 dendrimers (molecular weight 39.8 kD) had a longer blood half-life than the lower generation Gd-dendrimers because the particle size of standard Gd-G4 dendrimers is at the threshold of effective renal filtration [17]. Irrespective of dose, Gd-G5 through Gd-G8 dendrimers maintained steady state blood concentrations over a minimum of 2 hours because particle sizes of these generations of Gd-dendrimers are clearly above the threshold of effective renal filtration [17]. As a result of the long blood half-lives, Gd-G5 and Gd-G6 were able to slowly extravasate across the BBTB of even the smallest gliomas that we studied. Based on these findings, we conclude that it may be possible to effectively deliver permeable nanoparticles with long blood half-lives across a minimally compromised BBTB, including across the BBTB of the microvasculature supplying emerging malignant glioma colonies.

To verify that only permeable functionalized dendrimers with long blood half-lives accumulate within malignant glioma cells, we infused rhodamine B labeled Gd-G5 dendrimers and rhodamine B labeled Gd-G8 dendrimers to separate groups of rats. The dose of rhodamine B Gd-dendrimers was 0.06 mmol Gd/kg bw, since in pilot experiments we observed that the anesthetic effect of isoflurane was potentiated at the 0.09 mmol Gd/kg bw rhodamine B Gd-dendrimer dose [59,60]. Fluorescence microscopy of RG-2 glioma specimens demonstrated extensive subcellular localization of rhodamine B Gd-G5 dendrimers, confirming that functionalized G5 dendrimers accumulate within malignant glioma cells, due to long blood half-lives.

We observed with both fluorescence microscopy and dynamic contrast-enhanced MRI that there was some accumulation of rhodamine B Gd-G8 dendrimers in RG-2 gliomas (Figure 5C and 5E), as well as some non-selective accumulation of rhodamine B Gd-G5 and rhodamine B Gd-G8 dendrimers in tumor-free brain regions (Additional file 5). We suspect that rhodamine B labeled Gd-G5 and Gd-G8 dendrimers are toxic to the BBTB in addition to the otherwise healthy blood-brain barrier. This toxicity is likely due to the introduction of additional positive charge to the Gd-dendrimer surface from the attachment of rhodamine B, a cationic and lipophilic fluorescent dye [61-64]. Therefore, the extravasation of rhodamine labeled nanoparticles [26,65] and other charged nanoparticles [66-69] across the barrier may be from direct charge induced damage to endothelial cells of the barrier and disruption of the barrier. Our proposed mechanism for the increased barrier permeation of rhodamine labeled Gd-dendrimers is analogous to the mechanism recently proposed by Herce and Garcia [70,71] for the movement of cell-penetrating peptides across cell membranes. We plan to clarify, in the future, with additional *in vivo *imaging experiments, the relationship between charge on the dendrimer surface and disruption of the blood-brain barrier.

## Conclusion

In this study, we identified the precise physiologic upper limit of blood-brain tumor barrier pore size, and demonstrated that nanoparticles of diameters smaller than this upper limit can effectively traverse the pores of the blood-brain tumor barrier; in addition, we validated the importance of prolonged nanoparticle blood half-life for the effective accumulation of nanoparticles within brain tumor cells. Therefore, based on these findings, we conclude that effective drug delivery across the BBTB of malignant gliomas, and potentially the BBB of other neuropathologies, can be accomplished with non-toxic nanoparticles that are smaller than 11.7 to 11.9 nm in diameter and have prolonged blood half-lives.

In the broadest sense, our findings will serve as general guidelines, for the future design and development of multifunctional transvascular delivery devices, based on nanoparticles (i.e. liposome-, quantum dot-, or iron oxide-based) and biological particles (i.e. antibody- or viral-based), that are particularly effective at crossing the diseased BBB and accumulating in neuropathologic tissues.

## Competing interests

The authors declare that they have no competing interests.

## Authors' contributions

HS conceptualized, designed, and supervised the overall study; performed the dynamic contrast-enhanced MRI experiments, analyzed the data, interpreted the overall study results, and prepared the manuscript. ASK performed the dynamic contrast-enhanced MRI experiments, analyzed the data, and assisted with the preparation of the manuscript. HW synthesized and performed the preliminary characterization of the functionalized dendrimers. KRB assisted with the confocal fluorescence microscopy experiments. SHF performed the initial dynamic contrast-enhanced MRI experiments. KS assisted with the preparation of the manuscript. AAS characterized the higher generation functionalized dendrimers by electron microscopy. SA performed the statistical data analysis. CMW assisted with the synthesis of the functionalized dendrimers. MAA assisted with the characterization of the higher generation functionalized dendrimers by electron microscopy. RDL supervised the electron microscopy-based characterization of the functionalized dendrimers. GLG supervised the synthesis and preliminary characterization of the functionalized dendrimers, and contributed to the design of the overall study. MDH conceptualized, designed, and supervised the confocal fluorescence microscopy experiments; assisted with the interpretation of the overall study results, and prepared the manuscript.

## Supplementary Material

Additional file 1 **Amount of Gd-PAMAM dendrimer infused per Gd dose**. Click here for file

Additional file 2 **Gd-dendrimer residence time within the extravascular extracellular brain tumor space increases with increasing dendrimer generation at 0.09 mmol Gd/kg body weight dose**. At the 0.03 mmol Gd/kg bw dose, changes in the concentration profiles of Gd-G1 (left), Gd-G2 (middle) and Gd-G3 (right) are not evident. 0.09 mmol Gd/kg body weight dose, Gd-G1 (n = 5), Gd-G2 (n = 6), Gd-G3 (n = 6). 0.03 mmol Gd/kg bw dose, Gd-G1 (n = 6), Gd-G2 (n = 5), Gd-G3 (n = 5). Error bars represent standard deviation weighted for total tumor volume and are shown once every five minutes for clarity. Average tumor concentration curves are weighted with respect to total tumor volume within the respective dendrimer generation. Click here for file

Additional file 3 **Gd-dendrimers do not enter the normal brain extravascular space due to the normal blood-brain barrier**. Shown are dynamic contrast-enhanced MRI concentration curves at the 0.09 mmol Gd/kg body weight dose. Gd-G1 (n = 5) and Gd-G5 (n = 6) as representative examples of low and high dendrimer generation behavior. Error bars represent standard deviation and are shown once every five minutes for clarity. Average concentration curves are from normal brain tissue volumes of 9 mm^3 ^per brain. Click here for file

Additional file 4 **Physical properties of rhodamine B Gd-PAMAM dendrimers**. Click here for file

Additional file 5 **Rhodamine labeled Gd-G5 and rhodamine labeled Gd-G8 dendrimers enter the normal brain extravascular space across the normal blood-brain barrier**. Shown are dynamic contrast-enhanced MRI concentration curves of rhodamine Gd-dendrimers at a 0.06 mmol Gd/kg body weight dose and Gd-dendrimers at a 0.09 mmol Gd/kg body weight dose. A) Rhodamine Gd-G5 (n = 6), Gd-G5 (n = 6). B) Rhodamine Gd-G8 (n = 2), Gd-G8 (n = 6). Error bars represent standard deviation and are shown once every five minutes for clarity. Average concentration curves are from normal brain tissue volumes of 9 mm^3 ^per brain. Click here for file
