# Supplementary material for: Effective transvascular delivery of nanoparticles across the blood-brain tumor barrier into malignant glioma cells
Source: J Transl Med. 2008 Dec 18;6:80. doi: 10.1186/1479-5876-6-80 (PMC2639552; doi:10.1186/1479-5876-6-80)
Supplement: Additional file 1 — Amount of Gd-PAMAM dendrimer infused per Gd dose. [file 1479-5876-6-80-S1.pdf]

Additional file 1 – Amount of Gd-PAMAM dendrimer infused per Gd dose

Gd-dendrimer generation	0.03 mmol Gd/kg bw (mg Gd-dendrimer)	0.06 mmol Gd/kg bw (mg Gd-dendrimer)	0.09 mmol Gd/kg bw (mg Gd-dendrimer)
G1	32	—	94
G2	32	—	96
G3	37	—	110
Lowly conjugated G4	38	—	115
G4	39	—	118
G5	40	—	119
Rhodamine B G5	—	96	—
G6	40	—	119
G7	39	—	116
G8	46	—	139
Rhodamine B G8	—	101	—
