# Supplementary figures and images for: Effective transvascular delivery of nanoparticles across the blood-brain tumor barrier into malignant glioma cells
Source: J Transl Med. 2008 Dec 18;6:80. doi: 10.1186/1479-5876-6-80 (PMC2639552; doi:10.1186/1479-5876-6-80)

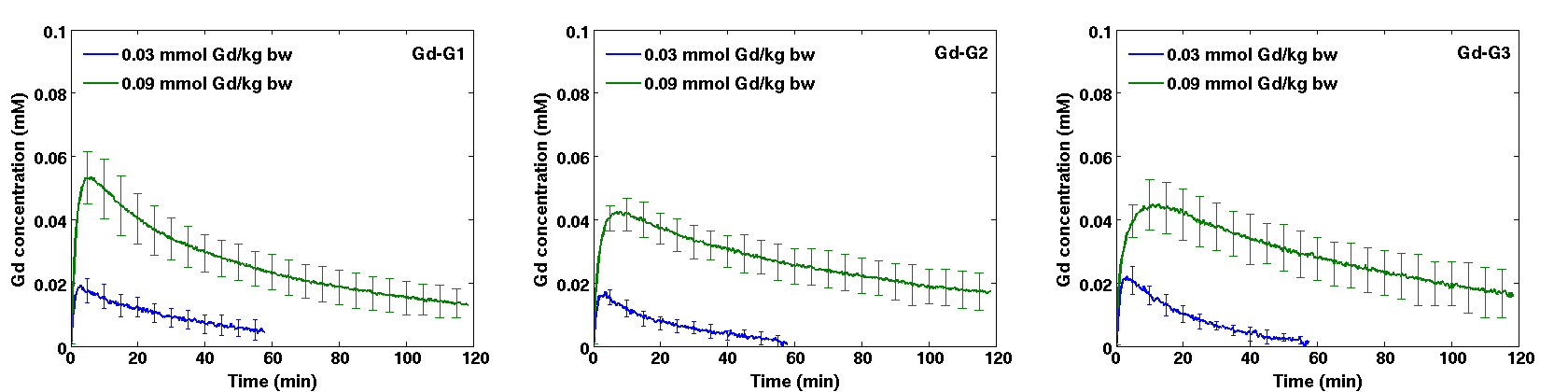

Supplement: Additional file 2 — Gd-dendrimer residence time within the extravascular extracellular brain tumor space increases with increasing dendrimer generation at 0.09 mmol Gd/kg body weight dose. At the 0.03 mmol Gd/kg bw dose, changes in the concentration profiles of Gd-G1 (left), Gd-G2 (middle) and Gd-G3 (right) are not evident. 0.09 mmol Gd/kg body weight dose, Gd-G1 (n = 5), Gd-G2 (n = 6), Gd-G3 (n = 6). 0.03 mmol Gd/kg bw dose, Gd-G1 (n = 6), Gd-G2 (n = 5), Gd-G3 (n = 5). Error bars represent standard deviation weighted for total tumor volume and are shown once every five minutes for clarity. Average tumor concentration curves are weighted with respect to total tumor volume within the respective dendrimer generation. [file 1479-5876-6-80-S2.jpeg]

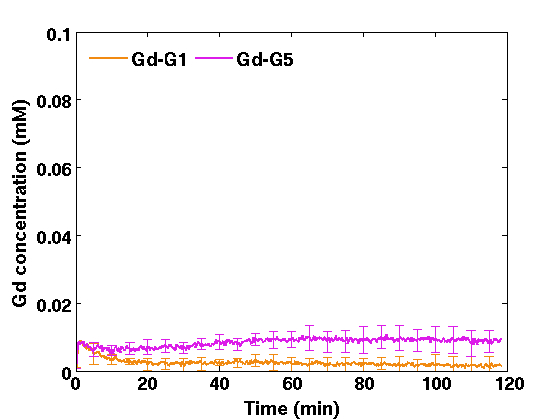

Supplement: Additional file 3 — Gd-dendrimers do not enter the normal brain extravascular space due to the normal blood-brain barrier. Shown are dynamic contrast-enhanced MRI concentration curves at the 0.09 mmol Gd/kg body weight dose. Gd-G1 (n = 5) and Gd-G5 (n = 6) as representative examples of low and high dendrimer generation behavior. Error bars represent standard deviation and are shown once every five minutes for clarity. Average concentration curves are from normal brain tissue volumes of 9 mm3 per brain. [file 1479-5876-6-80-S3.jpeg]

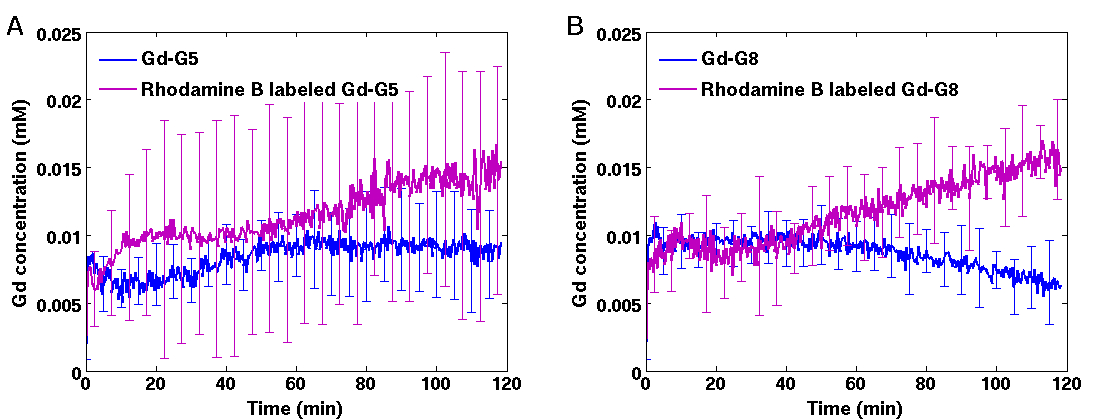

Supplement: Additional file 5 — Rhodamine labeled Gd-G5 and rhodamine labeled Gd-G8 dendrimers enter the normal brain extravascular space across the normal blood-brain barrier. Shown are dynamic contrast-enhanced MRI concentration curves of rhodamine Gd-dendrimers at a 0.06 mmol Gd/kg body weight dose and Gd-dendrimers at a 0.09 mmol Gd/kg body weight dose. A) Rhodamine Gd-G5 (n = 6), Gd-G5 (n = 6). B) Rhodamine Gd-G8 (n = 2), Gd-G8 (n = 6). Error bars represent standard deviation and are shown once every five minutes for clarity. Average concentration curves are from normal brain tissue volumes of 9 mm3 per brain. [file 1479-5876-6-80-S5.jpeg]
