# Supplementary material for: Effective transvascular delivery of nanoparticles across the blood-brain tumor barrier into malignant glioma cells
Source: J Transl Med. 2008 Dec 18;6:80. doi: 10.1186/1479-5876-6-80 (PMC2639552; doi:10.1186/1479-5876-6-80)
Supplement: Additional file 4 — Physical properties of rhodamine B Gd-PAMAM dendrimers. [file 1479-5876-6-80-S4.pdf]

Additional file 4 – Physical properties of rhodamine B Gd-PAMAM dendrimers

Rhodamine B Gd-dendrimer generation	No. terminal amines	Molecular weight# (kD)	Gd-DTPA Conjugation %	Rhodamine B Conjugation %	Molar Relaxivity (s/mM)
G2	16	11.2	42.7	6.6	n/a
G5	128	75.0	36.5	7.9	9.2
G8	1024	540‡	31.2	6.6	9.2

#obtained from Dendritech, Inc.

‡measured by ADF STEM
